# Oral *Eupolyphaga sinensis* extract promotes lumbar interbody fusion by enhancing vascularization of cartilage endplate

**DOI:** 10.3389/fsurg.2025.1652156

**Published:** 2025-08-29

**Authors:** Ruixin Zhen, Jiaqi Li, Shaorong Li, Han Wu, Wei Zhang

**Affiliations:** ^1^ Department of Spine Surgery, First Hospital of Hebei Medical University, Shijiazhuang, China; ^2^ Department of Spine Surgery, Affiliated Hospital of Chengde Medical University, Chengde, China; ^3^ Department of Spine Surgery, Hebei Medical University Third Hospital, Shijiazhuang, China

**Keywords:** *Eupolyphaga sinensis*, cartilage endplate, angiogenesis, interbody fusion, spine, rabbit

## Abstract

**Objective:**

This study aims to investigate the effect of oral *Eupolyphaga sinensis* (ES) extract on intervertebral fusion in a rabbit model.

**Methods:**

A rabbit lumbar lateral interbody fusion model was established. Different treatments were administered to each group, including the control group (CON), oral ES extract group (ES), interbody fusion group (IBF) and interbody fusion combined with oral ES group (IBF/ES). Ten weeks after surgery, micro-CT was used to assess intervertebral bone fusion. Bone trabecula parameters, including bone volume fraction (BV/TV), trabecular number (Tb.N) and trabecular thickness (Tb.Th), were measured. Histological analyses, including Masson and HE staining, were used to evaluate angiogenesis and bone growth in the endplate.

**Results:**

The micro-CT at the 10th postoperative week showed significant bone tissue regeneration and stable fusion in the IBF/ES groups. Fusion scores, BV/TV, Tb.N and Tb.Th were significantly higher in the IBF/ES groups compared to the other groups. In addition, Masson and HE staining indicated evident vascular ingrowth and new bone formation after oral ES extract treatment. Among the four groups, the IBF/ES groups showed the most significant bone formation and the best fusion effect.

**Conclusion:**

This study suggested that oral ES extract after spine surgery can significantly enhance the effectiveness and success rate of lumbar lateral fusion surgery by promoting endplate vascularization and bone formation.

## 1 Introduction

Lumbar degenerative disc disease is a common disabling condition that often leads to back pain, leg pain, and spinal deformities (1). When conservative treatment fails, radiculopathy caused by disc herniation is often treated surgically through lumbar interbody fusion (2). Spinal fusion is a definitive method for treating progressive spinal deformities, instability, and certain spinal infections or tumors (3). At present, there are several surgical methods for spinal fusion, the main difference lying in the surgical approach to the intervertebral disc space. Lateral lumbar interbody fusion (LLIF) directly enters the intervertebral disc laterally through the retroperitoneal space, which can reduce the dissection of the spine and paravertebral muscles. Compared to posterior lumbar interbody fusion (PLIF), LLIF provides more graft space, reduces surgical trauma and intraoperative bleeding, which is more beneficial to postoperative recovery (4–6). Despite these advantages of LLIF, it is still challenging to achieve the interbody fusion. For example, nonunion or postoperative pseudarthrosis is still a major concern (7).

Exploring efficient and convenient fusion strategies is crucial to improving the effectiveness of LLIF. Autologous bone, which does not trigger an immune response, can be used as a scaffold for the growth of blood vessels and bone tissue and provides some biological factors beneficial to osteogenesis. This is considered as the gold standard for interbody fusion (8, 9). However, harvesting autologous bone is accompanied by the risk of surgical complications and surgical pain, which limits the application in LLIF. Previous studies have shown that combining some bone biomaterials with bone morphogenetic protein (BMP) can significantly improve lumbar fusion rate (7, 10). However, excessive use of BMP would lead to adverse events, such as soft tissue swelling, local inflammation, aseptic cyst formation, osteolysis, implant displacement, ectopic bone formation, retrograde ejaculation and radiculitis (11–13). The failure rate of LLIF is between 7%–20% (14–16). In addition to the strategy of using autogenous bone or bone biomaterials during operation, postoperative medication can also improve the fusion effect. Recently, it was reported that in the posterolateral spinal fusion model of rabbits, intramuscular injection of abaloparatide combined with spinal fusion surgery significantly increased the rate and effect of fusion (17, 18). Postoperative drug therapy can achieve significant therapeutic effects and offers higher safety, providing a promising strategy to improve lumbar fusion rate.

In recent years, with the development of bone tissue engineering (BTE), an increasing number of researchers have begun to analyze various bioactive components which are derived from traditional Chinese medicine (TCM) and use them to treat orthopedic diseases. For example, *Eupolyphaga sinensis* (ES) is a beneficial medicinal insect, commonly used in TCM, having the function of treating fractures. ES belongs to Eupolyphaga, which is widely distributed in Asia such as Thailand, India, Malaysia, and China (19). In TCM, ES is described as having properties of invigorating blood circulation, removing blood stasis, and healing bones and tendons. Due to its accessibility and efficacy, it is widely used in folk medicine for treating orthopedic conditions in China. Previous studies have shown that ES significantly enhances vascular regeneration and promotes bone regeneration (20, 21). At the cellular level, ES can promote the differentiation of rat bone marrow mesenchymal stem cells (BMSCs) towards osteoblasts by regulating BMP (22). From both folk application and scientific research perspectives, it is evident that ES has the ability to promote bone regeneration. This suggests its potential use for enhancing lumbar fusion, although its research and application in spinal surgery are relatively limited at present.

In LLIF, a cage and bone grafts are implanted into the intervertebral space to maintain intervertebral height and provide a scaffold for bone growth. During the fusion process, blood vessels and osteoblasts from cartilage endplate grow into intervertebral space with the support of the graft and cage leading to bone bridging and interbody fusion. Therefore, it is an ideal method to enhance the biological activity of endplate through adjunctive drug therapy based on surgical strategy to promote interbody fusion. The purpose of this study is to enhance the activity of blood vessels and bone cells in the endplate by using ES, thereby improving the lumbar fusion rate.

## 2 Methods

### 2.1 Preparation of ES extract

**Scheme 1:**
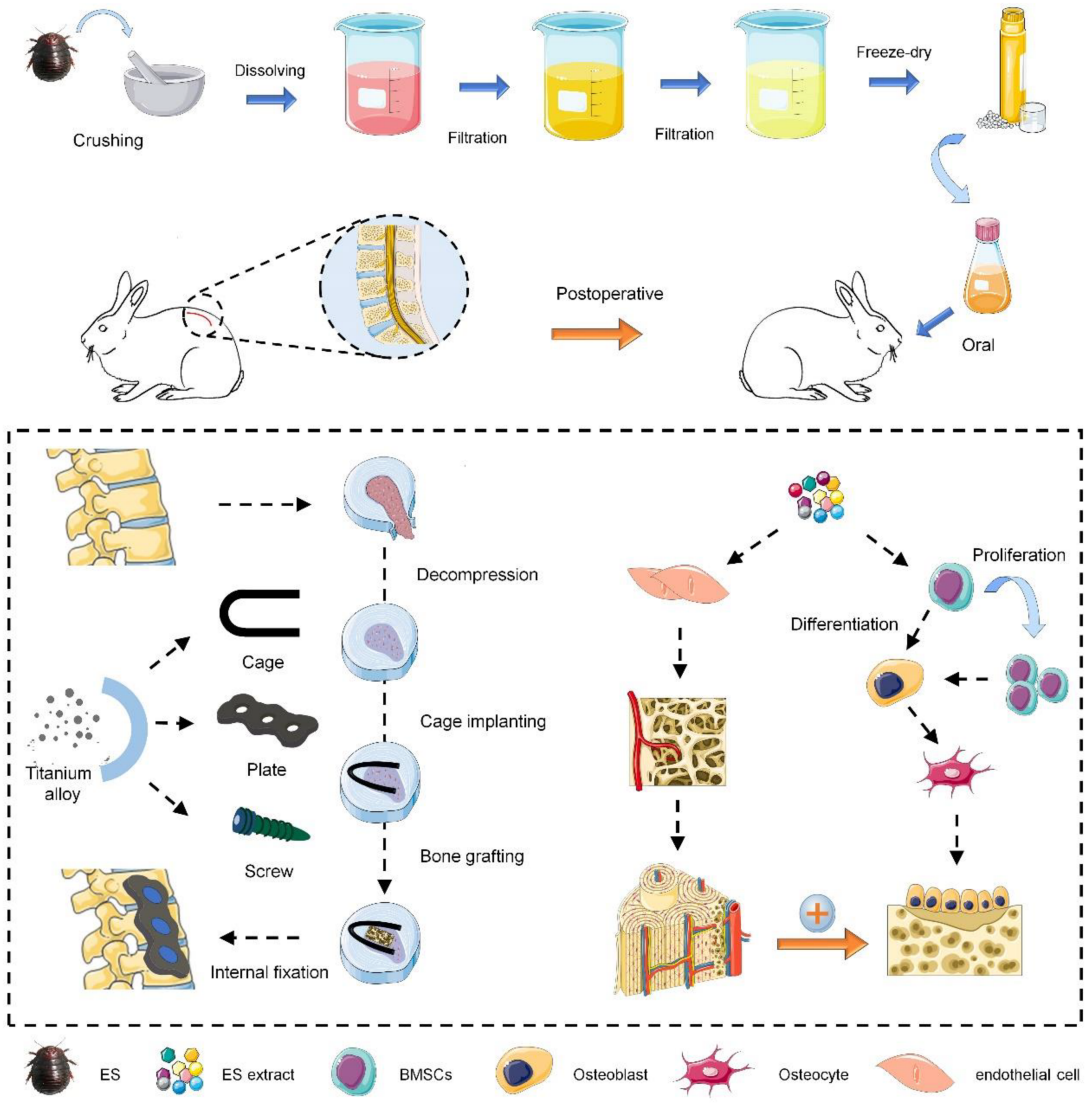
Schematic of the current study design.

As shown in Scheme 1, the preparation process of ES extract is as follows. Whole *Eupolyphaga sinensis* (Bozhou medicinal market, Anhui Province) was crushed and passed through a 40-mesh sieve to remove impurities. A total of 100 g ES powder was added to 800 ml of distilled water to prepare an aqueous solution, which was boiled for 40 min and then filtered to obtain the first filtrate. Repeat this process for the residue to collect the second filtrate. The combined filtrates were centrifuged at 8,000 rpm for 20 min, and the supernatant was freeze-dried to obtain the purified ES extract powder. The powder was dissolved in distilled water to make a solution of 5 mg/ml. The total mass of raw ES material was recorded as W_1_, and the final mass of extract powder was W_2_. The extraction rate was calculated as follows:

$$\mathrm{Product}\;\mathrm{ratio}\;(\text{\%})=W_{2}/\;W_{1}\;\times 100\text{\%}$$

### 2.2 HPLC

The changes in the ES extract after 3 months at room temperature were analyzed using high-performance liquid chromatography (HPLC). The detection conditions were as follows. Stationary phase: a TMC18 column (150 mm × 4.6 mm, 5 μm) was maintained at 30°C. The mobile phase flowed at a rate of 0.5 ml/min. The injection volume was set to 10 μl. Detection was performed at 254 nm, and the total running time was 10 min. After the procedure, the data was collected and compared.

### 2.3 Animal model

The animal study adheres to ARRIVE guidelines and has been approved by the Ethics Committee of the Affiliated Hospital of Chengde Medical University (NO.CYFYLL2023244). Twenty-four male Japanese white rabbits (2.0–2.5 kg) were used in the study and randomly divided into four groups (*n* = 6): control group (CON), oral ES group (ES), interbody fusion group (IBF), and interbody fusion combined with oral ES group (IBF/ES). In brief, anesthesia was induced with 5% isoflurane and maintained with 2.5%–3% isoflurane. The rabbits were placed in the right lateral position, and a 5-cm longitudinal incision was made in the left waist to expose the iliac crest, then the iliac bone was harvested, crushed, and used as bone graft. A longitudinal incision was made in the lumbar dorsal fascia to expose the deep paraspinal muscles, and the muscles were directly dissected to expose the lateral surface of the L4/5 intervertebral disc. After removing the nucleus pulposus and cartilage endplate using specialized tools, a cage and autogenous iliac bone were implanted, followed by fixation of adjacent vertebrae using steel plates and pedicle screws. Finally, the incision was washed with saline, and the muscle and fascia were sutured in layers. In the CON and ES groups, only the nucleus pulposus and cartilage endplate were removed, without implanting the cage and autologous bone. After the surgery, the incision was bandaged with sterile gauze and penicillin (40,000 U/kg) was injected into muscle for 3 days to prevent infection. General condition, wound healing, and nutritional status of the rabbits were monitored and recorded every day. Starting on the third day post-surgery, ES extract was administered in ES group and IBF/ES group at a dose of 160 mg/kg/day, while the same amount of water was administered in the CON group and IBF group. The dosage was adjusted every two weeks according to the weight change till the end of the experiment. All rabbits were euthanized at the 10th week post-surgery, and lumbar specimens were collected and kept in 4% paraformaldehyde for fixation.

### 2.4 Micro-CT

At the 10th postoperative week, the lumbar specimen was scanned and analyzed by Micro-CT (Bruker Skyscan1176, Switzerland). The parameters used for scanning were 50 kV, 500 mA, scanning mode (360 rotation), exposure time (250 ms), and image resolution of 1,024 × 1,024 pixels. Then Three-dimensional reconstruction of the images was performed using NRECON and CTVOX software. According to the CT results, fusion scores were evaluated by two blind observers. Bone volume to total volume ratio (BV/TV), trabecular thickness (Tb.Th), and trabecular number (Tb.N) were quantitatively analyzed.

### 2.5 Histological analysis

The specimens were fixed in 4% paraformaldehyde for 48 h, decalcified in 10% EDTA at 4°C, and embedded in paraffin. Sagittal sections (5 μm thick) were cut and stained with hematoxylin-eosin (HE) and Masson's. The angiogenesis and bone growth of endplate were observed under light microscope (DSX 500; Olympus Corporation, Japan).

### 2.6 Statistical analysis

All data was presented as mean ± SD, and statistical analysis was performed using SPSS 26.0 software. Data was analyzed using a two-tailed *t*-test (for comparisons between two groups) or one-way ANOVA (for comparisons among multiple groups) to determine the significance of differences at *p < 0.05, **p < 0.01, ***p < 0.001.

## 3 Results

### 3.1 Characterization of ES

**Figure 1:**
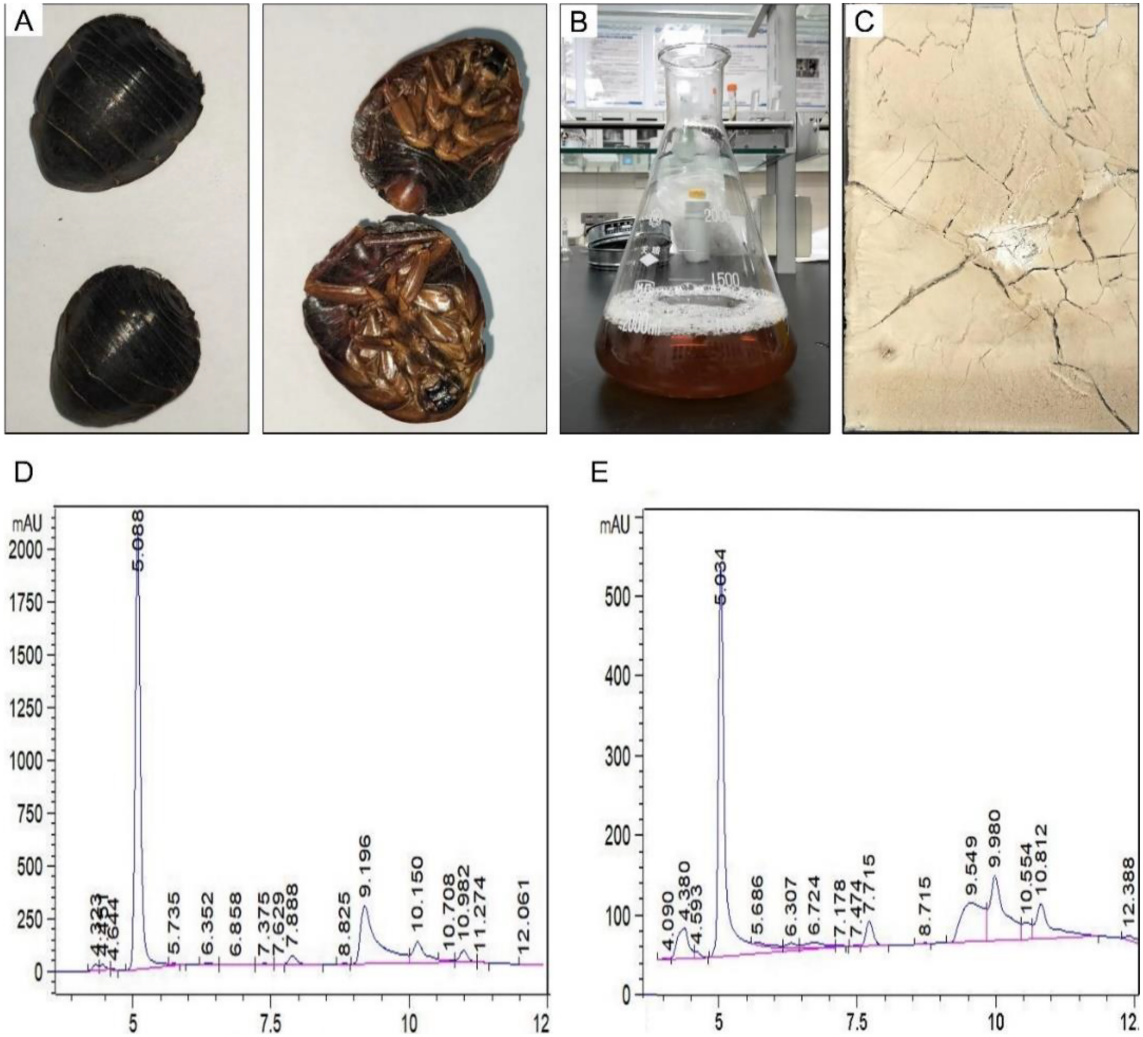
Characterization analysis of *Eupolyphaga sinensis* (ES) extract. **(A)** Whole insect of ES. **(B)** Filtrate of ES extract. **(C)** Freeze-dried powder of ES extract. HPLC analysis of ES extract before **(D)** and after **(E)** 3 months of storage (The vertical coordinates are different due to the different concentrations before and after).

At room temperature, whole ES was used as raw material to extract effective components (Figure 1A). After filtration and distillation, the extract filtrate was obtained (Figure 1B) and the final product was light yellow powder (Figure 1C). Approximately 16.20 g freeze-dried powder was finally obtained from the initial 100 g raw materials, yielding about 16.20% as calculated by the formula. In the preparation process, most impurities and poorly water-soluble components in whole ES were removed, which helps to avoid some side effects compared to direct decoction. In order to verify the drug stability during postoperative medication, the peak changes of the ES extract before and after storage for 3 months were analyzed by HPLC. As shown in Figures 1D,E, the distribution of absorption peaks was consistent before and after 3 months, indicating that the ES extract powder can be stably stored at room temperature for at least 3 months, which ensures the stability of its pharmaceutical composition and efficacy during the experimental process and clinical medication.

### 3.2 ES promotes interbody fusion

**Figure 2:**
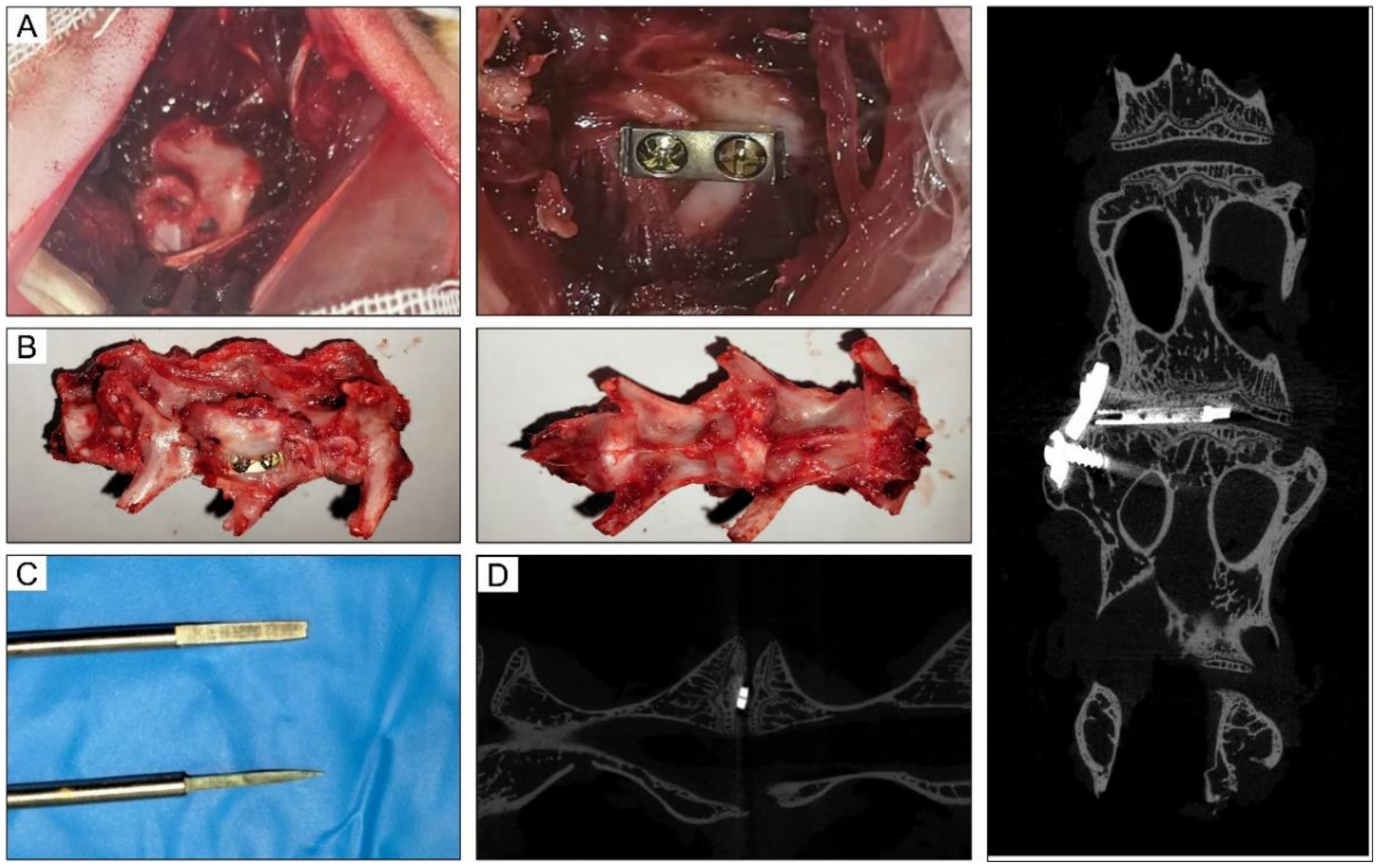
Intraoperative and imaging pictures of rabbit lumbar fusion model. **(A)** Intraoperative picture of L4-5 fusion process. **(B)** Lumbar fusion specimen after operation. **(C)** Customized tools used for rabbit fusion models. **(D)** The immediate micro-CT images after operation.

Rabbit models of lumbar fusion surgery were used in this study. To establish this model, the nucleus pulposus was firstly removed. Next, a titanium cage was implanted, and a steel plate was fixed to the adjacent vertebral body (Figure 2A). At the 10th postoperative week, the intervertebral space with interbody fusion in the IBF/ES group did not show bone gap (Figure 2B). Manual palpation revealed detectable motion at the fusion site. To establish this rabbit model, we customized some surgical tools (Figure 2C). These tools were designed to remove the nucleus pulposus from the narrow lumbar intervertebral space in rabbits, avoiding damage to the internal structures of the spinal canal. An immediate micro-CT scan after operation showed that the cage was well positioned and fixed (Figure 2D).

**Figure 3:**
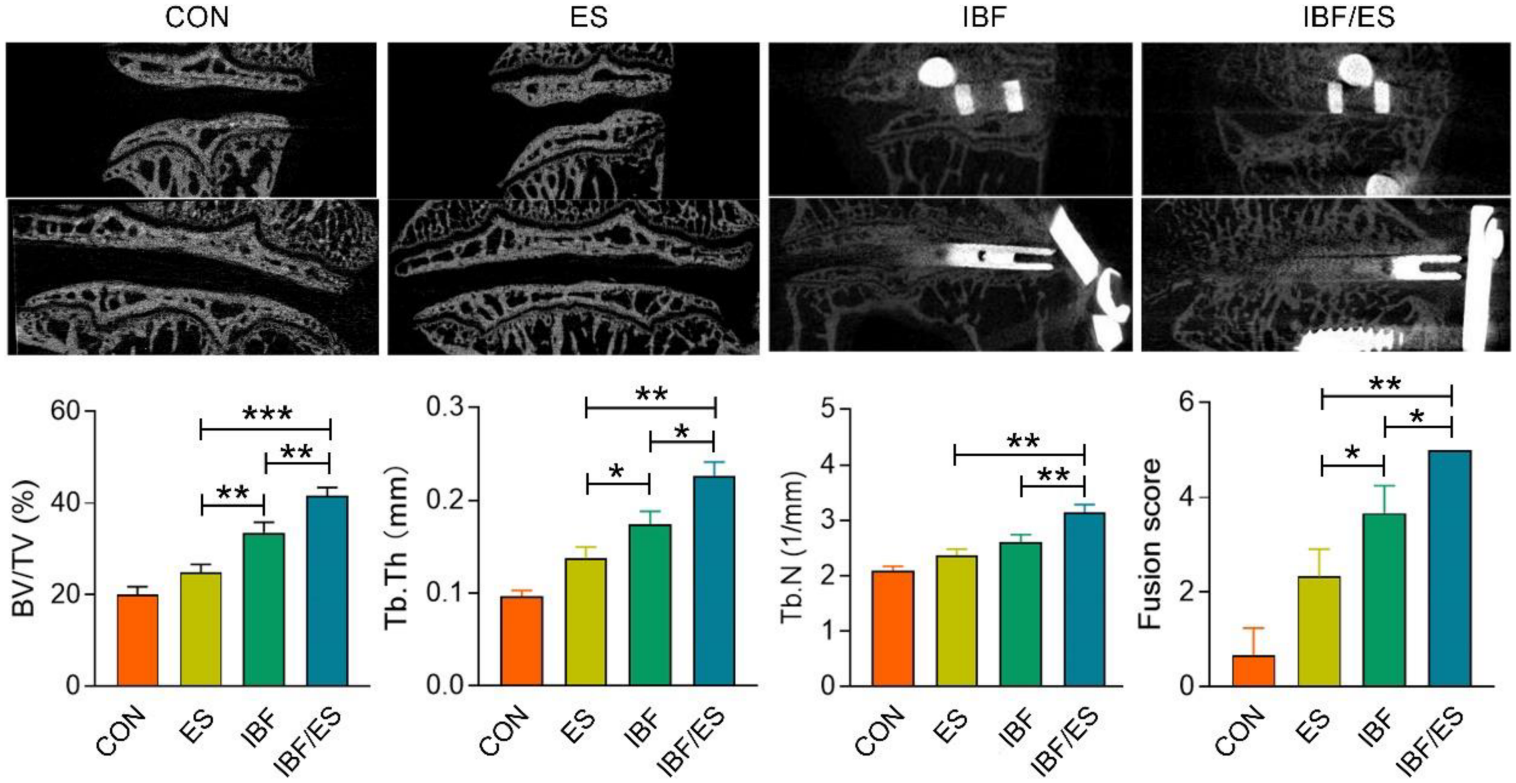
Micro-CT analysis of the intervertebral region at 10 weeks after operation. A rabbit lumbar lateral interbody fusion model was established. Different treatments were administered to each group, including the control group (CON), oral *Eupolyphaga sinensis* (ES) extract group, interbody fusion group (IBF) and interbody fusion combined with oral ES group (IBF/ES). Ten weeks later, micro-CT was conducted. *n* = 6, *P < 0.05; **P < 0.01, ***P < 0.001.

The results of Micro-CT for each group are presented in Figure 3. No significant differences were observed between the CON and ES groups in the three-dimensional reconstructed images. The laminae of these two groups were complete and the intervertebral height was reduced. In IBF group, the intervertebral space displayed heterogeneous signals and recognizable endplate boundary. In IBF/ES group, there was a clear trabecular connection in the intervertebral space, the bone bridge between the intervertebral space and the endplate disappeared, and the boundary of the endplate was unrecognizable. The fusion score, BV/TV, Tb.N and Tb.Th were further analyzed. In the CON, ES, IBF, and IBF/ES groups, the BV/TV were 20.113 ± 1.370, 24.835 ± 1.489, 33.552 ± 1.848, and 41.512 ± 1.594, respectively, showing significant differences among all groups (*P* < 0.05). The trabecular thicknesses were 0.097 ± 0.005, 0.138 ± 0.009, 0.174 ± 0.011, and 0.226 ± 0.013, respectively, with significant differences between groups (*P* < 0.05). The trabecular numbers were 2.097 ± 0.058, 2.363 ± 0.095, 2.601 ± 0.117, and 3.145 ± 0.115, respectively, which were significantly different among the groups, but not between the ES group and the IBF group. The fusion scores were 0.667 ± 0.117, 2.333 ± 0.471, 3.667 ± 0.471, and 5 ± 0.000, respectively, showing significant differences between groups (*P* < 0.05). Overall, the IBF/ES group had significantly higher fusion scores, Tb.Th, Tb.N, and BV/TV compared with the other three groups (*P* < 0.05).

### 3.3 ES promotes cartilage endplate angiogenesis and bone regeneration

**Figure 4:**
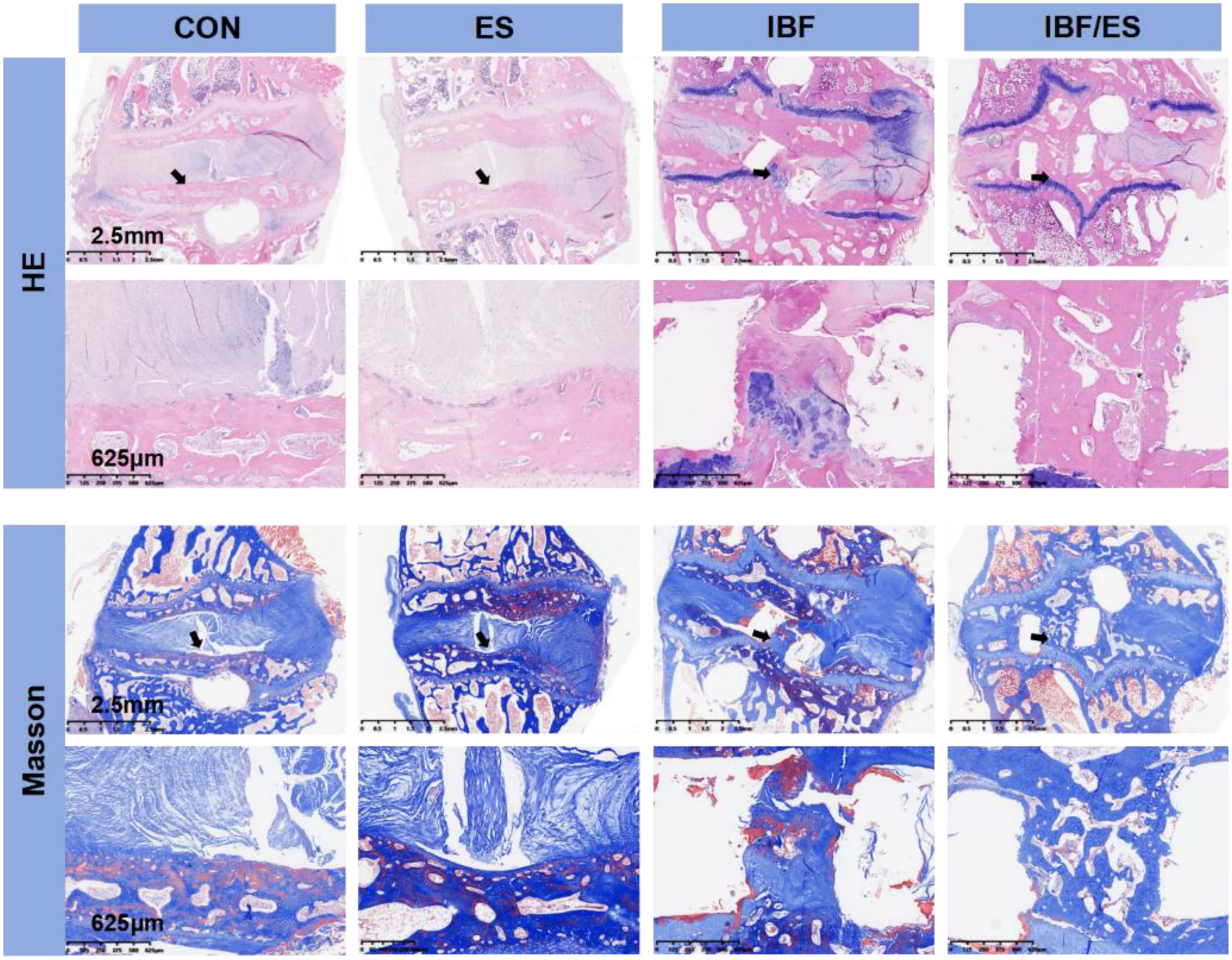
He and Masson staining of the intervertebral region at 10th week after operation. A rabbit lumbar lateral interbody fusion model was established. Different treatments were administered to each group, including the control group (CON), oral *Eupolyphaga sinensis* (ES) extract group, interbody fusion group (IBF) and interbody fusion combined with oral ES group (IBF/ES). Ten weeks later, samples were collected. Masson and HE staining was performed. *n* = 6.

At the 10th postoperative week, HE staining and Masson's trichrome staining are shown in Figure 4. The IBF/ES group showed a complete bony connection between the upper and lower endplates, suggesting that the combination of cage, internal fixation and oral ES achieved the best therapeutic effect. Blood vessels in cartilage endplate region of the CON group and the IBF group were significantly less compared with the ES group and the IBF/ES group, indicating that oral ES significantly increased the blood vessel regeneration in the endplate region. Additionally, in the ES group, a small amount of bone tissue was seen extending from the endplate into the intervertebral space, indicating that the increased blood supply in the endplate region promoted intervertebral bone regeneration. Masson staining further showed that there was significant trabecular formation in and around the cage in the IBF/ES group while there was no significant trabecular structure formation in the IBF group. Similarly, the ES group displayed bony processes in the endplate area, although the absence of a cage and robust internal fixation likely limited the extent of structural fusion.

## 4 Discussion

In the historical development of traditional Chinese medicine (TCM), the application of ES and its derivatives in the treatment of orthopedic diseases can be traced back to the Eastern Han Dynasty nearly 2,000 years ago. Classic Chinese medicine, including Synopsis of the Golden Chamber and Compendium of Materia Medica, clearly recorded the safety and effectiveness of oral ES in treating tendon and bone injuries (23). In addition, some studies related to TCM also show that ES has high safety. Previous reports show that ES has a high safety profile in animal research, and an LD50 exceeding 10 g/kg in rats (24). Although ES has a high safety threshold, its effective therapeutic concentration has not been fully studied. Li et al. found that administering ES at 1,000 mg/kg/day significantly promoted the healing of mandibular fracture in rabbits (25). In this study, the raw material dosage of ES was set at 1,000 mg/kg/day, and the actual dosage was 162 mg/kg/day based on the extraction rate of 16.2%. In the previous clinical application and research, ES and its preparations were made into decoction by boiling and eaten immediately. However, this method is affected by variables such as boiling conditions, making dosage control difficult.

In our study, ES extract powder was prepared by freeze-drying, which can form uniform and adjustable drug concentration after dissolution. It is verified that ES powder can be stored at room temperature for at least 3 months, ensuring consistency of composition and efficacy during long-term use. During the experimental period, the freeze-dried ES powder was reconstituted into a 5 mg/ml solution and administered based on each rabbit's weight so that the dose of the extract can be accurately controlled and the influence of dose error on the experimental results can be reduced. Clinically, the drug concentration can be adjusted according to the individual weight difference between rabbits and humans to reduce the total volume.

At present, the extraction methods of ES primarily include water extraction and alcohol extraction. Studies have shown that ES water extract can promote the proliferation of human endothelial cells, whereas alcohol extract inhibit the proliferation of endothelial cells (20, 26). The major components of ES include amino acids, proteins, bioactive peptides, polysaccharides, lipids, alkaloids, nitrogen-containing compounds, nucleotides, fat-soluble vitamins, and inorganic substances. The differences between water and alcohol extraction are largely determined by the solubility characteristics of these components. Components with inhibitory effects on tumor cell proliferation, such as polysaccharides, oleic acid, and unsaturated fatty acids, are more efficiently extracted by alcohol. Components promoting angiogenesis and bone regeneration, such as bioactive peptides, alkaloids, and inorganic substances, are more efficiently extracted using water. ES has diverse biological functions, and different extraction methods can be used to obtain different active components according to practical therapeutic needs (27). However, comprehensive research on the complete chemical composition of ES remains limited and warrants further investigation.

This study is the first to investigate the relationship between endplate changes and interbody fusion using a rabbit interbody fusion model. Previous studies have shown that cages and bone graft serve as scaffolds and growth factors for intervertebral bone growth, thus improving the fusion rate (7). To further clarify the factors that influence interbody fusion, this study compared and analyzed the respective effects of drugs and cage between groups. Compared with the CON group, the ES group exhibited improved bone tissue parameters, likely due to the ability of ES extract which enhances endplate angiogenesis and increases intervertebral blood supply. Wei et al. reported that the culture medium containing ES extract promoted the proliferation of endothelial cells and bone marrow mesenchymal stem cells—two key cell types involved in bone tissue repair (20–22). Abundant local blood supply provides sufficient nutrients and growth factors for bone growth while removing metabolic wastes. The blood supply of intervertebral region decreases with the aging of the body, which makes the promotion of vascular ingrowth become increasingly important for interbody fusion (28). In addition, the ability of ES to promote the proliferation and osteogenic differentiation of bone marrow mesenchymal stem cells is more beneficial to enhance interbody fusion. However, due to the lack of intervertebral support in ES group, the growth of blood vessels and bone tissue into intervertebral space was hindered, resulting in the formation of bony spurs. During the fusion process, the cage and internal fixation device provide stable space support and scaffolding between the upper and lower endplates, thereby facilitating the sustained ingrowth of blood vessels and bone cells into the intervertebral space. This structural support explains why stable bone connection is finally achieved only in IBF/ES group.

Interbody fusion is characterized by “the formation of bone connections between the upper and lower endplates, including bone connections within the endplate and bone bridge connections at the endplate edge” (29, 30). Previous studies reported that the average time required for spinal fusion for small animals such as rabbits and rats was 6–8 weeks (31, 32). However, these studies employed transverse process fusion models rather than interbody fusion. Because the intervertebral region is subjected to higher mechanical stress and has relatively poor blood supply, the fusion process is typically slower and less efficient. Therefore, a more clinically relevant interbody fusion model was adopted in our study, and this model was established according to the fusion cage and graft materials currently used in clinic. Our previous research demonstrated that the lateral lumbar fusion in rabbits achieved a fusion rate of 91.7% at approximately 12 weeks postoperatively (33). However, in the current study, the IBF/ES group achieved bone fusion by the 10th week after operation, and the fusion rate was 100%. These findings suggest that ES extract significantly accelerates and enhances process of interbody fusion.

Cage subsidence and endplate collapse are common complications following lumbar fusion. When the cage sinks, it directly reduces intervertebral height, compromises anterior spinal support, and alters adjacent soft tissue structure, which may ultimately impair the result of surgical decompression. Liu et al. reported a case in which the cage subsidence led to a reduction in intervertebral height and ligament laxity, further compressing nerve roots and led to recurrence (34). Therefore, preventing cage subsidence is crucial for maintaining sagittal balance and optimizing surgical outcomes. Our study not only addressed the issue of improving fusion rates but also the prevention of cage subsidence. Oral ES extract after operation promoted angiogenesis and enhanced bone density in adjacent endplate regions, which may help prevent cage subsidence. The results of micro-CT showed significant increases in BV/TV, Tb.Th, and Tb.N in the adjacent vertebrae of the ES and IBF/ES groups, indicating a beneficial effects of ES extract on surrounding vertebral structures. Finally, the improvement of trabecular structure and the increase of adjacent vertebrae density are beneficial to prevent the loss of intervertebral height caused by cage subsidence.

One possible pathway using oral ES extract is that it can be used as an adjunctive therapy in human lumbar fusion. From a clinical standpoint, lumbar fusion typically involves the use of autologous bone grafts or allogeneic materials to promote bone regeneration. This study demonstrated that oral administration of ES extract significantly promotes angiogenesis and bone regeneration within the intervertebral region. Thus, oral ES extract has the potential to be routinely employed postoperatively for human lumbar fusion, even replacing the need for bone grafts. From the aspect of BTE, although the pharmacological composition, functions and mechanisms of ES are complex, its pronounced therapeutic effects warrant further investigation. Therefore, the future research will focus on the basic research of ES and the development of local application methods of ES drugs from combined BTE.

## 5 Conclusion

In conclusion, this study suggested that oral ES extract after spine surgery can significantly enhance the effectiveness and success rate of lumbar lateral fusion surgery by promoting cartilage endplate vascularization and bone formation. This study provides preclinical evidence supporting the use of ES extract to promote interbody fusion and highlights its potential application in treatment of bone-related diseases.

## Data Availability

The raw data supporting the conclusions of this article will be made available by the authors, without undue reservation.
